# Dense fine speckled antinuclear antibody patterns: Clinical correlations and implications

**DOI:** 10.46439/rheumatology.5.027

**Published:** 2025

**Authors:** Smarika Sapkota, John Crosson, Michael D. Evans, Londyn Robinson, Adam Lord, Benjamin Kofoed, Jerry A. Molitor

**Affiliations:** 1 Division of Rheumatology, Department of Medicine, National Jewish Health, Denver, CO 80206, USA; 2 Department of Lab Medicine and Pathology, University of Minnesota, Minneapolis, MN 55455, USA; 3 Biostatistical Design and Analysis Center, Clinical and Translational Science Institute, University of Minnesota, Minneapolis, MN 55414, USA; 4 Division of Rheumatology, University of Washington, Seattle, WA 98195, USA; 5 Department of Family Medicine and Community Health, University of Minnesota, Minneapolis, MN 55455, USA; 6 Primary Care Track, Department of Internal Medicine, Emory University, Atlanta, GA 30322, USA; 7 Division of Rheumatology, Department of Medicine, University of Minnesota, Minneapolis, MN 55455, USA

**Keywords:** Anti-nuclear antibodies (ANA), Systemic Autoimmune Rheumatic Diseases (SARD), Dense fine speckled (DFS) pattern, Anti-DFS70 antibodies

## Abstract

**Background/Purpose:**

The significance of Dense Fine Speckled (DFS) pattern Anti-nuclear antibodies (ANA) by indirect mmunofluorescence (IIF) is unclear in the existing research. We aimed to investigate associations between positive ANA with DFS pattern and multiple autoimmune and rheumatic conditions.

**Methods:**

This retrospective study analyzed datasets from patients tested for ANA between August 2017 and August 2019. Comparisons were made between diagnostic categories and diseases for patients with negative ANA, positive ANA (any pattern), and positive ANA with DFS pattern. Relative risk (RR) was calculated for diagnostic categories and individual diseases.

**Results:**

Of 13,845 ANA results, 65.8% were negative and 34.2% were positive, including 4.6% with DFS pattern. Among ANA positive DFS pattern patients, 10.6% had inflammatory arthritis, 20.6% had fibromyalgia/chronic pain syndrome/chronic fatigue, 13.3% had SARD and only 2.2% had atopic disorder. Comparing ANA positive other patterns and DFS pattern, specific diagnoses like seropositive RA, SLE, SSc, and UCTD were lower among ANA positive DFS pattern. Similarly, diagnoses of Raynaud’s with SSc or UCTD were lower in DFS pattern patients compared to ANA positive with other patterns. The rate of other diagnoses including seronegative RA, IIM, SS, autoimmune thyroid disorder, and autoimmune hepatitis did not differ between other patterns and DFS pattern.

**Conclusion:**

The presence of DFS pattern cannot indiscriminately exclude the presence of SARD or rheumatic disease, as diagnoses including seronegative RA, IIM, SS, autoimmune thyroid disorder, or autoimmune hepatitis did not differ among ANA positive with other patterns and DFS pattern.

## Introduction

Anti-nuclear antibodies (ANA) play a crucial role in diagnosing Systemic Autoimmune Rheumatic Diseases (SARD) [1,2]. ANAs can be positive in healthy populations and in patients with non-autoimmune diseases, potentially causing unnecessary anxiety and increased healthcare resource utilization [2–5]. Previous studies indicate that some healthy individuals with positive ANA exhibit a dense fine speckled (DFS) pattern [3,6], often linked to anti-DFS70 antibodies- later identified as antibodies to lens epithelium-derived growth factor (LEDGF/p75) [3,7–9]. Based on existing research, the presence of monospecific anti-DFS70 antibodies, defined as anti-DFS 70 antibodies without other common Extractable nuclear antigen (ENA) antibodies, may reliably exclude SARD [1]. Emerging studies have examined the clinical associations of ANA positivity with the DFS pattern. Antibodies against LEDGF/p75, which present as a DFS pattern, have been identified in patients with interstitial cystitis, atopic dermatitis, asthma, Vogt-Koynagi-Harada syndrome, and Sjogren’s disease (SS) [8,9].

As a screening test, ANA should be performed using indirect immunofluorescence (IIF) which is considered as the gold standard [10], to leverage the diagnostic implications of the patterns and titers determined with IIF. ANA IIF test is being used frequently by primary care providers for patients suspected of having SARD. A positive result typically leads to a referral to rheumatology and further testing for ENA antibodies to assess the likelihood of SARD. This algorithm usually has its downside; in the absence of clinical features associated with SARD and with negative ENA results, diagnostic resolution is often lacking, resulting in anxiety among the patients. We recognized this gap in current clinical practice and aimed to further explore the clinical significance of ANA positivity with DFS pattern, as this pattern is generally considered a negative predictor of SARD when no other ENA antibodies are present.

## Methods

This study was conducted at the University of Minnesota Medical Center, where results were obtained from samples that were sent to the laboratory for IIF assay for ANA. The Euroimmune IIF platform was utilized for this test. IRB approval (#00010845) was granted by the University of Minnesota.

This retrospective study analyzed a dataset containing detailed information on patients tested for ANA via IIF between August 2017 to August 2019 was used. The dataset included medical record numbers, serum collection dates, and demographic information such as gender, age at data collection, race, and ethnicity. Data on ANA results, titers and pattern types were also recorded, with a positive ANA titer defined as ≥ 1:80. The final diagnosis associated with ANA testing was obtained.

For the subset of patients who tested ANA-positive with a DFS pattern, detailed demographic and clinical information was extracted from electronic medical records (EMR). For each patient, we collected data on age, sex, specialty of the ordering provider, symptoms leading to ANA order, past medical history (PMH) and final diagnosis related to ANA testing from EMR. We utilized the existing data on 50% of the ANA positive patients with DFS patterns from our previous observational study, which examined part of the same cohort [11]. Due to impracticality of manually reviewing charts for all patients with ANA tests during the study period, we collected data from chart reviews or EMR only on patients with DFS pattern.

Using the dataset that included final diagnosis information for all patients tested for ANA, we categorized the diagnosis into four disease categories: inflammatory arthritis, SARD, atopic disorder, or fibromyalgia/chronic fatigue syndrome/chronic pain syndrome. These categories were selected based on findings from our previous observational study, which revealed a higher prevalence of ANA positivity with a DFS pattern within these groups [11].

The inflammatory arthritis disease category encompassed seropositive rheumatoid arthritis (RA), seronegative RA, juvenile idiopathic arthritis (JIA), ankylosing spondylitis (AS), psoriatic arthritis (PsA), and inflammatory bowel disease (IBD)-associated arthropathy. The SARD category included systemic lupus erythematosus (SLE), systemic sclerosis (SSc), mixed connective tissue disease (MCTD), inflammatory myopathies (IIM) such as dermatomyositis (DM) and polymyositis (PM), Sjogren’s disease (SS), and undifferentiated connective tissue disorder (UCTD). Diagnoses of fibromyalgia, chronic pain syndrome, and chronic fatigue syndrome were grouped together due to significant overlap in clinical features. Atopic disorders were classified separately and included atopic dermatitis, allergic rhinitis, urticaria, and asthma. Additionally, diagnoses of psoriasis, Raynaud’s phenomenon (RP), autoimmune thyroid disorders, and autoimmune hepatitis were considered individually. Diagnostic information was derived from ICD-10 codes used for billing. The relative risk (RR) of the four disease categories and individual diseases was calculated for patients who were ANA-negative, ANA positive (any patterns), and ANA-positive with a DFS pattern. To validate our findings, we conducted a chart review to confirm the final diagnoses of patients with a DFS pattern. Discrepancies in the total number of patients with ANA positivity and DFS patterns between Table 1 (data from chart review) and Tables 2 and 3 (data from the Laboratory Medicine dataset) arose because only patients with a final diagnosis obtained through ICD-10 codes in the Laboratory Medicine dataset were included for relative risk calculations. However, we successfully obtained diagnoses for all patients with ANA positive and DFS patterns through chart review.

### Statistical analysis

The occurrence of diseases within each test result category was summarized using counts and rates. Differences in disease rates between categories were reported as relative risks (RR) with 95% confidence intervals and compared using Fisher’s exact test. Analyses were conducted using R version 4.0.4, including epitools packaged version 0.5–10.1.

## Results

### Demographic and clinical characteristics of patients with ANA-positive DFS pattern

Table 1 summarizes the demographic and clinical characteristics of patients with ANA positivity and DFS pattern, based on an EMR review.

The mean age was 43.3 years, with a predominance of females (76.9%). ANA testing was primarily ordered by primary care providers (55.0%), followed by rheumatologists (12.2%). The most common symptom leading to ANA testing with DFS positivity was joint pain (54.6%), followed by fatigue (26.2%), and rash (21.4%). Usually patients had multiple symptoms prompting the test.

The most common medical history (PMH) included atopic disorders (22.9%), osteoarthritis (10.7%) and fibromyalgia/chronic pain/chronic fatigue syndrome (10.3%). Other PMH diagnoses, such as myocarditis, interstitial lung disease, and pericarditis, were also noted.

Final diagnoses confirmed by EMR review showed inflammatory arthritis (12.0%) as the most common, followed by fibromyalgia/chronic pain/chronic fatigue (11.5%). A total of 29 patients (4.1%) had RP, however the primary and secondary subsets were not differentiated. Nineteen (2.7%) patients were lost to follow-up and those patients were not classified under any disease categories, but their data were still included to calculate the demographic and clinical characteristics included in Table 1. Of the remaining patients, 49.6% did not have a diagnostic resolution that could be categorized into specific disease categories based on chart review.

### Disease diagnosis in categories among patients with ANA positive with DFS pattern

Table 2 shows the prevalence and RR of disease categories- such as inflammatory arthritis, SARD, chronic pain/fibromyalgia/chronic fatigue syndrome, and atopic disorders- among ANA-negative, ANA positive, and DFS-pattern ANA-positive patients, based on the laboratory dataset.

Among 13,845 patients tested for ANA over two years, 9,106 (65.8%) were ANA-negative, and 4,739 (34.2%) were ANA-positive, including those with DFS patterns. Among these 13,845 patients, 1,333 (9.6%) had inflammatory arthritis, 1,613 (11.7%) had SARD, 2,859 (20.7%) had chronic pain/fibromyalgia/chronic fatigue syndrome, and 457 (3.3%) had a diagnosis of atopic disorder. A total of 640 (4.6%) patients were ANA positive with a DFS pattern.

Table 2 also presents RR and confidence intervals. ANA-positive patients (any pattern) had a higher RR for inflammatory arthritis and SARD than ANA negative patients. However, no significant difference in RR was observed for chronic pain syndromes or atopic disorders between ANA-positive and ANA-negative groups. Comparisons between DFS pattern and other ANA-positive patterns showed lower RR for SARD in the DFS group (Table 3).

### Relative risk of individual diseases among patients with ANA positive and DFS pattern

Table 4 and 5 highlight the prevalence and RR of specific disease diagnoses.

When comparisons were made between patients who had positive and negative ANA, RR was found to be higher for RA in general for the positive group, in addition to SLE, IIM, SSc, SS, UCTD, RP, autoimmune thyroid disease, psoriasis, autoimmune hepatitis, and fibromyalgia.

For DFS pattern ANA-positive patients, RR was notably higher for RA, SLE, SS, UCTD, fibromyalgia, RP, and autoimmune thyroid disorders compared to ANA-negative patients. However when comparing DFS patterns with other ANA-positive patterns, RR was lower for total RA, seropositive RA, SLE, SSc, and UCTD but not significantly different for SS, fibromyalgia, seronegative RA, RP, IIM, autoimmune thyroid disorder, or autoimmune hepatitis.

Although SLE, seropositive RA, SSc, and UCTD were more common in non-DFS ANA-positive, other SARDs-including SS, IIM, seronegative RA, autoimmune thyroid disorder, and autoimmune hepatitis did not follow this trend.

### Relative Risk (RR) of Raynaud’s Phenomenon (RP) with UCTD or with SSc (Table 6)

Table 6 compares ANA-positive patterns in patients diagnosed with RP and either UCTD or SSc diagnoses. Our findings indicate that non DFS ANA positive patterns was more prevalent in RP patients with UCTD or SSc than the DFS pattern.

## Discussion

This study was conducted in part because rheumatologists frequently receive referrals from primary care providers for patients with a positive ANA, often displaying a DFS pattern. Previous research suggests that the DFS pattern with anti-DFS70 antibodies is a negative predictor of SARD; however, this finding has not been consistently replicated. Our goal was to clarify the clinical associations of the DFS pattern and differentiate it from other ANA patterns. Unlike previous studies that focused on anti-DFS70 antibodies, this study specifically examines the DFS pattern itself.

While the DFS pattern is often associated with anti-DFS70 antibodies [3,7,8], recent research suggests it may not be exclusively linked to DFS70 antibodies [12]. The study was done as such since DFS 70 antibody results were unavailable due to retrospective nature of the study. Additionally, DFS 70 antibody is still not widely used or available and clinicians may still need to interpret the significance of the DFS pattern when encountering patients with positive ANA without information on DFS 70 antibody.

In this study, 34.2% of patients had a positive ANA, and 4.6% had a DFS pattern. These rates align with prior studies, where ANA positivity ranged from 11.6% to 42.4% [13,14] and DFS pattern positivity ranged from 3.6% to 5.2 % [14,15]. Among patients with a DFS pattern, 13.3% were diagnosed with SARD, consistent with previous findings (3.4% –17.6%) [14,15]. Additionally, 9.5% had RA, comparable to prior reports (12.3% – 27.2%) [13,16].

Compared to other ANA patterns, the DFS pattern was associated with a lower rate of SLE, SSc, and UCTD, which aligns with the known correlation of homogeneous patterns with SLE and nucleolar patterns with SSc. This is consistent with prior research comparing the DFS and homogenous patterns in SARD diagnoses [14]. However, the prevalence of IIM, SS, autoimmune thyroid disorder, and autoimmune hepatitis did not differ significantly between DFS and other ANA patterns. This contrasts with the prior research where DFS 70 antibody known to present as a DFS pattern in ANA testing, has been considered as a negative predictor of autoimmune diseases in general [3,6,9].

In our study, 5.9% of patients with a DFS pattern were diagnosed with SS, a higher rate than previously reported (3.5%–4.4%) [17,18]. While no significant difference was found between DFS and other ANA patterns, SS was significantly more common in ANA-positive patients than in ANA-negative individuals. Based on this study, the presence of a DFS pattern cannot be used exclusively to exclude SS as a diagnosis among patients with a positive ANA. This conclusion also concurs with the recommendation from the international consensus on ANA patterns (ICAP) that a panel looking at antibodies to extractable nuclear antigens (ENAs) should be performed following a positive ANA regardless of the pattern [1].

Raynaud’s phenomenon (RP) was more strongly associated with ANA positivity than ANA negativity. When stratifying patients with RP in those with UCTD or SSc, other ANA patterns were more prevalent than the DFS pattern. This suggests that the DFS pattern could be a potential negative predictor for the development of SSc in patients with RP. The presence of an ANA with a nucleolar pattern is relatively common for SSc spectrum disease, and the centromere pattern is also commonly seen in limited SSc [19,20]. These patterns are likely to be more representative of patients with positive ANA and diagnosis of SSc than DFS patterns. This adds to the relevance of the DFS pattern as a potential negative predictor for the diagnosis of SSc and UCTD especially when evaluating patients with RP.

A strength of this study is its validation of DFS pattern diagnoses through EMR review, cross-referenced with rheumatologist or primary care physician documentation. The EMR review identified 707 DFS pattern cases, while the databank only considered 640 patients during RR calculation, discrepancies were due to untraceable ICD-10 billing codes. Among DFS pattern patients, 10.6% were classified as having inflammatory arthritis based on ICD-10 codes, and 12.0% per EMR review. Similarly, SARD diagnoses were found in 7.9% based on EMR review and 13.3% based on ICD-10 codes. The ANA-positive cohort, used as a control, had significantly higher rates of lupus, SSc, SS, and UCTD, aligning with expectations based on the existing knowledge.

The study’s large sample size (13,845 ANA-tested patients) is another strength, making it one of the most extensive studies in the field. It includes data from both academic and community practices affiliated with the university.

A key limitation is the reliance on billing codes for diagnoses, which may overrepresent autoimmune diseases and potentially skew the results. Although the rates of each disease and disease category are not significantly different, there seems to be an overrepresentation of SARD based on ICD-10 codes when compared to chart review. Additionally, the retrospective design limits control over patient-related factors that may influence results and introduce biases.

Future studies should focus on incorporating more specific diagnostic criteria and additional biomarkers to improve the accuracy of identifying SARD in ANA-positive patients. Expanding the study to include more diverse populations and geographic regions could enhance the generalizability of the findings. Additionally, conducting a thorough analysis of confounding factors, such as medications and comorbidities, could provide a clearer understanding of the DFS patterns. Future research with a prospective design is necessary to account for patient-related factors, which could reduce potential biases and strengthen the validity of the findings.

In conclusion, the DFS pattern alone should not be used to exclude SARD, as suggested in previous studies [3,6]. While patients with DFS patterns had lower rates of seropositive RA, SLE, SSc, and UCTD than ANA-positive patients with other patterns, the rates were comparable for SS, IIM, autoimmune thyroid disorder, autoimmune hepatitis, and seronegative RA. In patients with RP undergoing autoimmune evaluation, the DFS pattern may serve as a negative predictor for SSc. However, DFS pattern detection in ANA-positive patients is not sufficient to rule out autoimmune diseases. A thorough clinical assessment by a rheumatologist, including reflexive ENA and anti-DFS70 testing, remains essential for accurate diagnosis.

## Figures and Tables

**Table 1.** Baseline demographic and clinical characteristics of patients who had ANA positive with DFS pattern based on EMR review.

	Number
Total number	707
Mean age in years (± SD)	43.3 (± 18.6)
Gender (%)	
Female	544 (76.9)
Male	163 (23.1)
Specialty of ordering provider, N (%)	
Primary care	389 (55)
Rheumatology	86 (12.2)
Neurology	37 (5.2)
Dermatology	29 (4.1)
Cardiology	23 (3.3)
Pediatrics	23 (3.3)
Others	120 (17.0)
Symptoms that led to ordering of ANA, N (%)	
Rash	151 (21.4)
Joint pain	386 (54.6)
Fatigue	185 (26.2)
Myalgia/muscle weakness	136 (19.2)
Past medical history N (%)	
osteoarthritis	76 (10.7)
Inflammatory arthritis	58 (8.2)
Atopic disorder	162 (22.9)
Systemic Autoimmune Rheumatic disease	24 (3.4)
Fibromyalgia/chronic pain/chronic fatigue	73 (10.3)
Psoriasis	31 (4.9)
Raynaud’s phenomenon	43 (6.1)
Autoimmune thyroid disease	36 (5.1)
Autoimmune hepatitis	1 (0.1)
Other diagnosis not classified	7 (1.0)
Final diagnosis, N (%)	
Inflammatory arthritis	85 (12.0)
Atopic disorder	34 (4.8)
Systemic autoimmune rheumatic diseases (SARD)	56 (7.9)
osteoarthritis	52 (7.6)
Fibromyalgia/chronic pain/chronic fatigue	81 (11.5)
Psoriasis	15 (2.1)
Raynaud’s phenomenon	29 (4.1)
Autoimmune thyroid disorder	17 (2.4)
Autoimmune hepatitis	2 (0.3)

**Table 2.** Relative Risk of Diagnostic Categories between ANA positive, ANA positive with DFS pattern and ANA negative patients.

Disease categories	ANA negative	ANA positive	Relative risk ¶ (95% CI)	p-value	ANA positive with DFS pattern	Relative risk € (95% CI)	p-value
Total	**9,106 (65.8%)**	**4,739 (34.2%)**			**640 (4.6%)**		
Inflammatory arthritis, n (%)	**717 (7.9)**	**616 (13.0)**	**1.65 (1.49–1.83)**	**<0.001**	**68 (10.6)**	**1.35 (1.07–1.71)**	**0.02**
Systemic Autoimmune Rheumatic Diseases, n (%)	**678 (7.4)**	**935 (19.7)**	**2.65 (2.42–2.91)**	**<0.001**	**85 (13.3)**	**1.78 (1.44–2.2)**	**<0.001**
Chronic pain/fibromyalgia/chronic fatigue syndrome, n (%)	**1,846 (20.3)**	**1,013 (21.4)**	**1.05 (0.98–1.13)**	**0.13**	**132 (20.6)**	**1.02 (0.87–1.19)**	**0.84**
Atopic disorder, n (%)	**295 (3.2)**	**162 (3.4)**	**1.06 (0.87–1.27)**	**0.58**	**14 (2.2)**	**0.68 (0.4–1.15)**	**0.16**

¶ Relative risk of disease given positive ANA result relative to ANA negative

€ Relative risk of disease given positive ANA with DFS pattern relative to ANA negative

**Table 3.** Relative Risk of Diagnostic Categories between ANA positive with DFS pattern and ANA positive with other patterns.

Disease categories	ANA negative	ANA positive	Relative risk ¶ (95% CI)	p-value	ANA positive with DFS pattern	Relative risk § (95% CI)	p-value
Total	**9,106 (65.8%)**	**4,739 (34.2%)**			**640 (4.6%)**		
Inflammatory arthritis, n (%)	**717 (7.9)**	**616 (13.0)**	**1.65 (1.49– 1.83)**	**<0.001**	**68 (10.6)**	**0.79 (0.63– 1.01)**	**0.06**
Systemic Autoimmune Rheumatic Diseases, n (%)	**678 (7.4)**	**935 (19.7)**	**2.65 (2.42–2.91)**	**<0.001**	**85 (13.3)**	**0.64 (0.52–0.79)**	**<0.001**
Chronic pain/fibromyalgia/chronic fatigue syndrome, n (%)	**1,846 (20.3)**	**1,013 (21.4)**	**1.05 (0.98– 1.13)**	**0.13**	**132 (20.6)**	**0.96 (0.82–1.13)**	**0.64**
Atopic disorder, n (%)	**295 (3.2)**	**162 (3.4)**	**1.06 (0.87– 1.27)**	**0.58**	**14 (2.2)**	**0.61 (0.35– 1.04)**	**0.08**

¶ Relative risk of disease given positive ANA result relative to ANA negative

§ Relative risk of disease given positive ANA with DFS pattern relative to ANA positive with different pattern

**Table 4.** Relative Risk of Diagnoses between ANA positive, ANA positive with DFS pattern and ANA negative patients.

Diagnosis	ANA negative	ANA positive	Relative risk¶ (95% CI)	p-value	ANA positive with DFS pattern	Relative Risk € (95% CI)	p-value
Total	9,106	4,739			640		
Ankylosing spondylitis, n (%)	**61 (0.7)**	**44 (0.9)**	**1.39 (0.94–2.04)**	**0.10**	**6 (0.9)**	**1.4 (0.61–3.22)**	**0.45**
Rheumatoid arthritis, all, n (%)	**646 (7.1)**	**573 (12.1)**	**1.70 (1.53–1.90)**	**<0.001**	**61 (9.5)**	**1.34 (1.05–1.73)**	**0.03**
Seropositive RA, n (%)	**282 (3.1)**	**291 (6.1)**	**1.98 (1.69–2.33)**	**<0.001**	**20 (3.1)**	**1.01 (0.65–1.58)**	**0.91**
Seronegative RA, n (%)	**330 (3.6)**	**231 (4.9)**	**1.35 (1.14–1.60)**	**<0.001**	**32 (5.0)**	**1.38 (0.97–1.97)**	**0.08**
RA not specified, n (%)	**34 (0.4)**	**51 (1.1)**	**2.88 (1.87–4.44)**	**<0.001**	**9 (1.4)**	**3.77 (1.81–7.82)**	**0.002**
Psoriatic arthritis, n (%)	**35 (0.4)**	**27 (0.6)**	**1.48 (0.90–2.45)**	**0.14**	**3 (0.5)**	**1.22 (0.38–3.95)**	**0.74**
							
SLE, n (%)	**216 (2.4)**	**368 (7.8)**	**3.27 (2.78–3.86)**	**<0.001**	**24 (3.8)**	**1.58 (1.04–2.39)**	**0.04**
Idiopathic Inflammatory myositis, n (%)	**53 (0.6)**	**52 (1.1)**	**1.89 (1.29–2.76)**	**0.001**	**5 (0.8)**	**1.34 (0.54–3.35)**	**0.43**
Scleroderma/systemic sclerosis, n (%)	**98 (1.1)**	**161 (3.4)**	**3.16 (2.46–4.05)**	**<0.001**	**10 (1.6)**	**1.45 (0.76–2.77)**	**0.24**
Sjogren’s syndrome, n (%)	**260 (2.9)**	**358 (7.6)**	**2.65 (2.26–3.09)**	**<0.001**	**38 (5.9)**	**2.08 (1.49–2.89)**	**<0.001**
Undifferentiated connective tissue disease, n (%)	**154 (1.7)**	**295 (6.2)**	**3.68 (3.04–4.46)**	**<0.001**	**22 (3.4)**	**2.03 (1.31–3.15)**	**0.003**
Chronic fatigue syndrome, n (%)	**741 (8.1)**	**416 (8.8)**	**1.08 (0.96–1.21)**	**0.20**	**65 (10.2)**	**1.25 (0.98–1.59)**	**0.08**
Chronic pain, n (%)	**907 (10.0)**	**482 (10.2)**	**1.02 (0.92–1.13)**	**0.70**	**48 (7.5)**	**0.75 (0.57–1.00)**	**0.05**
Fibromyalgia, n (%)	**694 (7.6)**	**435 (9.2)**	**1.20 (1.07–1.35)**	**0.002**	**68 (10.6)**	**1.39 (1.10–1.76)**	**0.009**
							
Raynaud’s phenomenon, n (%)	**411 (4.5)**	**456 (9.6)**	**2.13 (1.87–2.42)**	**<0.001**	**57 (8.9)**	**1.97 (1.51–2.57)**	**<0.001**
Psoriasis, n (%)	**421 (4.6)**	**276 (5.8)**	**1.26 (1.09–1.46)**	**0.002**	**34 (5.3)**	**1.15 (0.82–1.61)**	**0.44**
Autoimmune thyroid disorder, n (%)	**1,104 (12.1)**	**690 (14.6)**	**1.20 (1.10–1.31)**	**<0.001**	**99 (15.5)**	**1.28 (1.06–1.54)**	**0.02**
Autoimmune hepatitis, n (%)	**31 (0.3)**	**39 (0.8)**	**2.42 (1.51–3.87)**	**<0.001**	**2 (0.3)**	**0.92 (0.22–3.83)**	**1.00**

¶ Relative risk of disease given positive ANA result relative to ANA negative

€ Relative risk of disease given positive ANA with DFS pattern relative to ANA negative

**Table 5.** Relative Risk of Diagnoses between ANA positive with DFS pattern and ANA positive with other patterns.

Diagnosis	ANA negative	ANA positive	Relative risk ¶ (95% CI)	p-value	ANA positive with DFS pattern	RELATIVE RISK § (95% CI)	p-value
Total	9,106	4,739			640		
Ankylosing spondylitis, n (%)	**61 (0.7)**	**44 (0.9)**	**1.39 (0.94– 2.04)**	**0.10**	**6 (0.9)**	**1.01 (0.43– 2.38)**	**1.00**
Rheumatoid arthritis, all, n (%)	**646 (7.1)**	**573 (12.1)**	**1.70 (1.53–1.90)**	**<0.001**	**61 (9.5)**	**0.76 (0.59– 0.98)**	**0.03**
Seropositive RA, n (%)	**282 (3.1)**	**291 (6.1)**	**1.98 (1.69– 2.33)**	**<0.001**	**20 (3.1)**	**0.47 (0.30– 0.74)**	**<0.001**
Seronegative RA, n (%)	**330 (3.6)**	**231 (4.9)**	**1.35 (1.14– 1.60)**	**<0.001**	**32 (5.0)**	**1.03 (0.72– 1.48)**	**0.84**
RA not specified, n (%)	**34 (0.4)**	**51 (1.1)**	**2.88 (1.87– 4.44)**	**<0.001**	**9 (1.4)**	**1.37 (0.67– 2.81)**	**0.41**
Psoriatic arthritis, n (%)	**35 (0.4)**	**27 (0.6)**	**1.48 (0.90– 2.45)**	**0.14**	**3 (0.5)**	**0.80 (0.24– 2.65)**	**1.00**
SLE, n (%)	**216 (2.4)**	**368 (7.8)**	**3.27 (2.78– 3.86)**	**<0.001**	**24 (3.8)**	**0.45 (0.30– 0.67)**	**<0.001**
Idiopathic Inflammatory myositis, n (%)	**53 (0.6)**	**52 (1.1)**	**1.89 (1.29–2.76)**	**0.001**	**5 (0.8)**	**0.68 (0.27–1.71)**	**0.54**
Scleroderma/systemic sclerosis, n (%)	**98 (1.1)**	**161 (3.4)**	**3.16 (2.46–4.05)**	**<0.001**	**10 (1.6)**	**0.42 (0.22–0.80)**	**0.005**
Sjogren’s syndrome, n (%)	**260 (2.9)**	**358 (7.6)**	**2.65 (2.26–3.09)**	**<0.001**	**38 (5.9)**	**0.76 (0.55–1.05)**	**0.11**
Undifferentiated connective tissue disease, n (%)	**154 (1.7)**	**295 (6.2)**	**3.68 (3.04–4.46)**	**<0.001**	**22 (3.4)**	**0.52 (0.34–0.79)**	**0.001**
Chronic fatigue syndrome, n (%)	**741 (8.1)**	**416 (8.8)**	**1.08 (0.96–1.21)**	**0.20**	**65 (10.2)**	**1.19 (0.92–1.52)**	**0.20**
Chronic pain, n (%)	**907 (10.0)**	**482 (10.2)**	**1.02 (0.92–1.13)**	**0.70**	**48 (7.5)**	**0.71 (0.53–0.94)**	**0.02**
Fibromyalgia, n (%)	**694 (7.6)**	**435 (9.2)**	**1.20 (1.07–1.35)**	**0.002**	**68 (10.6)**	**1.19 (0.93–1.52)**	**0.19**
Raynaud’s phenomenon, n (%)	**411 (4.5)**	**456 (9.6)**	**2.13 (1.87–2.42)**	**<0.001**	**57 (8.9)**	**0.91 (0.70–1.19)**	**0.56**
Psoriasis, n (%)	**421 (4.6)**	**276 (5.8)**	**1.26 (1.09–1.46)**	**0.002**	**34 (5.3)**	**0.90 (0.63–1.28)**	**0.65**
Autoimmune thyroid disorder, n (%)	**1104 (12.1)**	**690 (14.6)**	**1.20 (1.10–1.31)**	**<0.001**	**99 (15.5)**	**1.07 (0.88–1.30)**	**0.47**
Autoimmune hepatitis, n (%)	**31 (0.3)**	**39 (0.8)**	**2.42 (1.51–3.87)**	**<0.001**	**2 (0.3)**	**0.35 (0.08–1.43)**	**0.16**

¶ Relative risk of disease given positive ANA result relative to ANA negative

§ Relative risk of disease given positive ANA with DFS pattern relative to ANA positive with different pattern

**Table 6.** ANA results among patients with Raynaud’s Phenomenon (RP) with diagnosis of Systemic Sclerosis (SSc) or with Undifferentiated Connective Tissue Disease (UCTD).

Diagnosis	ANA negative	ANA positive	Relative Risk ¶ (95% CI)	P value	ANA positive with DFS pattern	Relative risk § (95% CI)	p-value	Relative risk € (95% CI)	p-value
Total	9,106	4,739			640				
RP+ UCTD, n (%)	33 (0.4)	107 (2.3)	6.23 (4.22–9.19)	<0.001	6 (0.9)	0.38 (0.17– 0.86)	0.01	2.59 (1.09– 6.15)	0.04
RP + SSc, n (%)	22 (0.2)	80 (1.7)	6.99 (4.36–11.19)	<0.001	4 (0.6)	0.34 (0.12– 0.92)	0.02	2.59 (0.89–7.48)	0.09

¶ Relative risk of disease given positive ANA result relative to ANA negative

§ Relative risk of disease given positive ANA with DFS pattern relative to ANA positive with different pattern

€ Relative risk of disease given positive ANA with DFS pattern relative to ANA negative

## Abbreviations:

ANA: Antinuclear Antibodies
IIF: Indirect Immunofluorescence
DFS: pattern- Dense Fine speckled pattern
RR: Relative Risk
SARD: Systemic Autoimmune Rheumatic Diseases
LEDGF/p75: Lens Epithelium Derived Growth Factor
ENA: Extractable Nuclear Antigens
EMR: Electronic Medical Records
PMH: Past Medical History
RA: Rheumatoid Arthritis
JIA: Juvenile Idiopathic Arthritis
AS: Ankylosing Spondylitis
PsA: Psoriatic Arthritis
IBD: Inflammatory Bowel Disease
SLE: Systemic Lupus Erythematosus
SSc: Systemic Sclerosis
MCTD: Mixed Connective Tissue Disease
IIM: Inflammatory Myopathies
DM: Dermatomyositi
PM: Polymyositis
SS: Sjogren’s Disease
UCTD: Undifferentiated Connective Tissue Disorder
RP: Raynaud’s Phenomenon
ICAP: International Consensus on ANA Pattern
DFS70 antibody: Anti-dense Fine Speckled 70 antibody
PsA: Psoriatic arthritis
IBD: Inflammatory bowel disease -associated arthropathy
